# Additional support for RCR: A validated article-level measure of scientific influence

**DOI:** 10.1371/journal.pbio.2003552

**Published:** 2017-10-02

**Authors:** B. Ian Hutchins, Travis A. Hoppe, Rebecca A. Meseroll, James M. Anderson, George M. Santangelo

**Affiliations:** 1 Office of Portfolio Analysis, Division of Program Coordination, Planning, and Strategic Initiatives, National Institutes of Health, Bethesda, Maryland, United States of America; 2 Division of Program Coordination, Planning, and Strategic Initiatives, National Institutes of Health, Bethesda, Maryland, United States of America; Walter and Eliza Hall Institute of Medical Research, Australia

In their comment, Janssens et al. [1] offer a critique of the Relative Citation Ratio (RCR), objecting to the construction of both the numerator and denominator of the metric. While we strongly agree that any measure used to assess the productivity of research programs should be thoughtfully designed and carefully validated, we believe that the specific concerns outlined in their correspondence are unfounded.

Our original article acknowledged that RCR or, for that matter, any bibliometric measure has limited power to quantify the influence of any very recently published paper, because citation rates are inherently noisy when the absolute number of citations is small [2]. For this reason, in our *iCite* tool, we have not reported RCRs for papers published in the calendar year previous to the current year [3]. However, while agreeing with our initial assertion that RCR cannot be used to conclusively evaluate recent papers, Janssens et al. also suggest that the failure to report RCRs for new publications might unfairly penalize some researchers. While it is widely understood that it takes time to accurately assess the influence that new papers have on their field, we have attempted to accommodate this concern by updating *iCite* so that RCRs are now reported for all papers in the database that have at least 5 citations and by adding a visual indicator to flag values for papers published in the last 18 months, which should be considered provisional [3]. This modified practice will be maintained going forward.

Regarding article citation rates of older articles, we have added data on the stability of RCR values to the “Statistics” page of the *iCite* website [4, 5]. We believe that these new data, which demonstrate that the vast majority of influential papers retain their influence over the period of an investigator’s career, should reassure users that RCR does not unfairly disadvantage older papers. Our analysis of the year-by-year changes in RCR values of National Institutes of Health (NIH)-funded articles published in 1991 reinforces this point (Fig 1). From 1992–2014, both on the individual level and in aggregate, RCR values are remarkably stable. For cases in which RCRs change significantly, the values typically increase. That said, we strongly believe that the potential for RCR to decrease over time is necessary and important; as knowledge advances and old models are replaced, publications rooted in those outdated models naturally become less influential.

**Fig 1:**
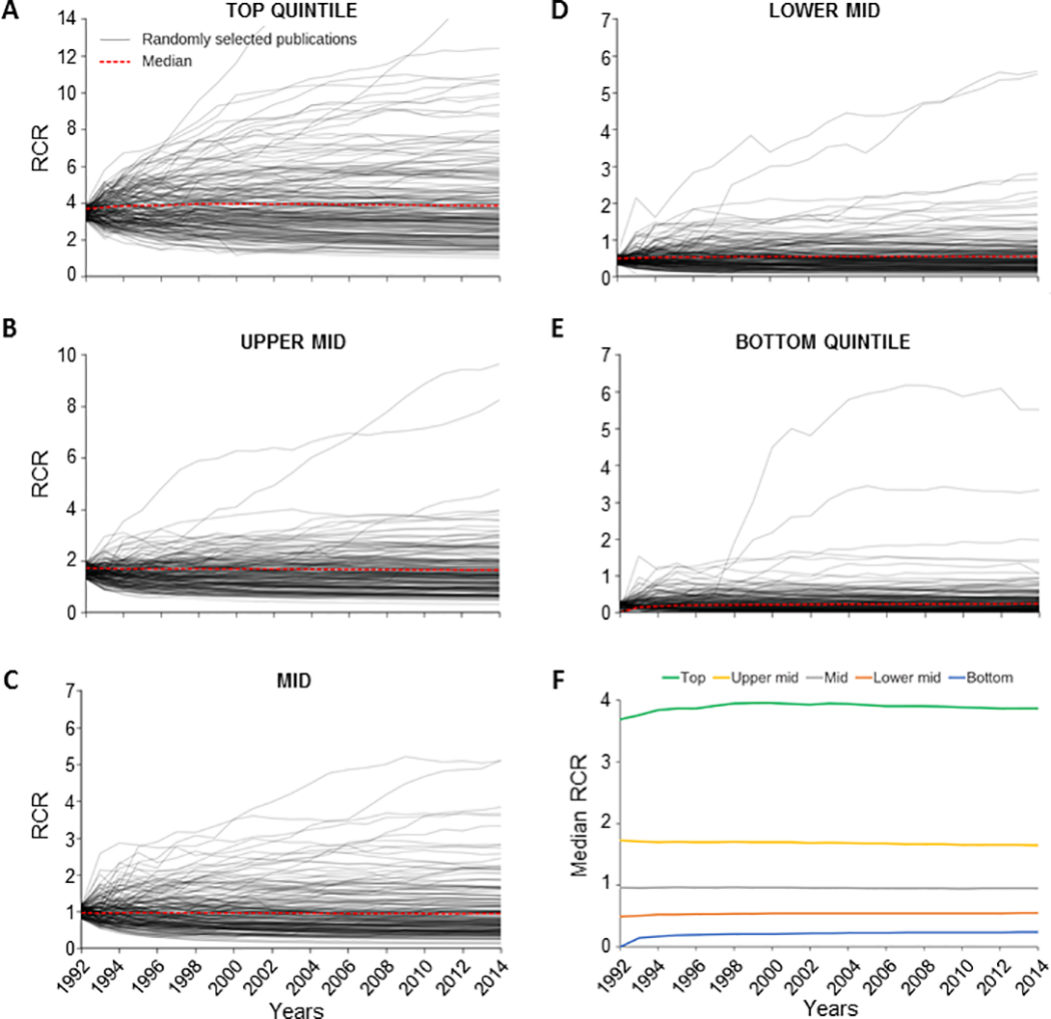
Stability of Relative Citation Ratio (RCR) over time. (A–E) Change in RCR over time was determined for individual National Institutes of Health (NIH)-funded articles published in 1991. Articles were assigned to quintiles based on their RCR values in the year after publication (1992); RCR in each subsequent year was calculated. For each quintile, 200 individual articles (gray lines) were chosen at random from the subset in which the 1992 values were within 10% of the median, and the resulting plots are shown. All values are actual and unsmoothed. In (A) through (E), the red line shows the median for the respective quintile. (A) Top quintile, (B) Upper mid quintile, (C) Mid quintile, (D) Lower mid quintile, (E) Bottom quintile, (F) Median RCR in each of the 5 quintiles for all NIH-funded articles published in 1991. See S1 Data.

The RCR denominator (the expected citation rate [ECR]) is calculated by aggregating the article citation rates of peer papers that have the same field citation rate (FCR) and are published in the same year as the article in the numerator. FCR, as noted by Janssens et al., is defined as the collective 2-year journal citation rate for all papers in the co-citation network of the article being evaluated. This calculation is conceptually distinct from that used in determining journal impact factors; rather than relying on journal of publication to define an article’s peers, our FCR considers all articles identified by publishing scientists as relevant comparators, regardless of the venue in which those articles appear. While acknowledging that there is almost certainly room for further theoretical advances in methods for defining the field of an individual article, we have demonstrated that co-citation networks better define an article’s field than discipline-specific journals such as *Blood*, *Genetics*, or the *Journal of Neuroscience* (Hutchins et al., Fig 2 [2]).

Janssens et al. object to these and other design choices we made in constructing RCR but do not test any alternative approaches that might improve the metric. For example, they criticize the choice of calculating FCRs based upon 2-year citation rates but do not offer an alternative. One testable suggestion—using only papers co-cited at least twice in the network—is acknowledged by the authors to involve unworkable trade-offs. Indeed, our preliminary testing suggests that this alternative design choice changes FCRs by no more than 5% but leads to a drastic reduction in the number of in-network articles (in agreement with the 80% estimate reported by Janssens and Gwinn [6]), thereby introducing finite number effects into the calculation of FCRs. The concomitant large increase in variance is essential to avoid, because an unstable denominator would have detrimental effects on the overall robustness of the RCR metric.

Finally, we take exception to Janssens et al.’s oversimplification that “the metric has become central in NIH’s grant management policy.” RCR is a validated metric of the influence of a publication and can be used as one measure of productivity. However, NIH considers many factors when making funding decisions, including public health burden, opportunities for scientific progress, workforce stability, and portfolio diversity [7].

We encourage further development of article-level bibliometrics and will enthusiastically adopt improved methodologies when their superior characteristics are demonstrated by a thorough analysis that includes at least some of the many tests that we presented in our original *PLOS Biology* paper. Until then, we remain convinced that RCR is the best available method for assessing the influence of research publications.

## Supporting information

S1 Data Data underlying the Fig 1 graphs. (XLSX)

## Abbreviations

ECR: expected citation rate
FCR: field citation rate
NIH: National Institutes of Health
RCR: Relative Citation Ratio
